# Postcoarctectomy syndrome: a contemporary systematic review

**DOI:** 10.3389/fsurg.2025.1518720

**Published:** 2025-11-17

**Authors:** Hector R. Martinez, Misael Salazar-Alejo, Ana Ballesteros-Suarez, Luis E. Perez-Martinez, Jose A. Moran-Guerrero, Rogelio E. Flores-Salcido, Vicente Jimenez-Franco, Silvia Cecilia Britton-Robles, Mario Benvenutti-Regato, Jose A. Figueroa-Sanchez

**Affiliations:** 1 Tecnologico de Monterrey, Escuela de Medicina y Ciencias de la Salud, Monterrey, Mexico; 2 Instituto de Neurología y Neurocirugía, Centro Médico Zambrano Hellion, TecSalud, San Pedro Garza García, Mexico; 3 Instituto de Cardiología y Medicina Vascular, Centro Médico Zambrano Hellion, TecSalud, San Pedro Garza García, México

**Keywords:** vascular medicine, vascular surgery, cardiothoracic surgery, adverse events, review—systematic

## Abstract

Postcoarctectomy syndrome is a serious but poorly understood complication that can occur following surgical or endovascular repair of coarctation of the aorta. This condition presents with severe abdominal pain, persistent hypertension, and gastrointestinal symptoms that can be life-threatening if not promptly recognized and treated. Despite being a known complication for decades, the underlying mechanisms of postcoarctectomy syndrome remain unclear, creating challenges for both early diagnosis and effective treatment. We present the case of a patient with postcoarctectomy syndrome and a systematic review analyzing the clinical presentation, diagnostic approach, and treatment strategies for this complication. We also conduct a systematic literature review, identify key knowledge gaps, and propose future research priorities to address them.

## 1 Introduction

Coarctation of the aorta (CoA) is a congenital heart disease characterized by a localized narrowing of the thoracic aorta. Most commonly, the coarctation occurs near the insertion of the ductus arteriosus, distal to the left subclavian artery. However, some patients present with more extensive narrowing affecting longer aortic segments or tortuous areas and frequently occurs alongside other congenital heart defects (1). Clinical signs and symptoms may include a systolic or continuous murmur, weak or absent femoral pulses, hypertension, and aortic dissection (2). Patients may also experience headaches, epistaxis, and develop brain aneurysms (3, 4).

The first successful intervention for CoA was independently reported in 1945 by Crafoord & Nylin (5) and Gross & Hufnagel (6). Since this breakthrough, treatment options have expanded to include minimally invasive approaches such as balloon angioplasty and stent placement (7). Despite successful and timely repair, patients remain at risk for several complications, including persistent hypertension, recoarctation, aortic dissection, and postcoarctectomy syndrome (PCS) (1, 2).

PCS is a serious complication characterized by abdominal pain and systemic hypertension following surgical or endovascular repair of CoA. While PCS typically develops within the first 7 days post-intervention, delayed presentation can occur weeks to months later. Patients may experience additional symptoms including vomiting, ileus, abdominal distension, fever, leukocytosis, and melena (3–7). Given that PCS can be life-threatening, clinicians must recognize this complication early and understand effective prevention and management strategies.

This review presents a representative case that demonstrates the clinical presentation, diagnostic approach, and treatment strategies for PCS. Additionally, we conduct a systematic review of current literature to synthesize existing knowledge, identify persistent knowledge gaps, and outline future research pathways to address them.

## 2 Case presentation

A Hispanic male in his late 40's initially presented 6 years prior to this report with an aneurysmal subarachnoid hemorrhage. During the aneurysm coiling procedure, digital subtraction angiography incidentally revealed CoA. After successfully managing the aneurysmal rupture, CT angiography of the aortic arch demonstrated a coarctation measuring 3.0 × 4.4 mm in diameter. At that time, CoA treatment was initially deferred to focus on neurological recovery and, despite medical advice, it was subsequently postponed based on the patient's preference (8).

Six years later, the patient returned with intermittent claudication. Blood pressure (BP) measurements revealed significant upper-to-lower extremity gradients and reduced ankle-brachial indices bilaterally. Despite treatment with 50 mg atenolol, 5 mg amlodipine, 160 mg valsartan, and 12.5 mg hydrochlorothiazide daily, the patient's BP remained elevated, averaging ∼150/106 mmHg. Endovascular aortic repair was decided.

Prior to the procedure, all antihypertensive medications except atenolol were discontinued. Invasive hemodynamic measurements demonstrated a 60-mmHg transcoarctation gradient. A covered stent (BeGraft, 10 × 40 mm) was successfully implanted at the coarctation site. Post-deployment imaging confirmed proper stent expansion, with no evidence of aortic dissection or rupture. Immediate postoperative BP improved to 102/60 mmHg, with no residual gradient across the stent. Following postoperative protocol, the patient was admitted to the ICU where he remained hemodynamically stable on continued atenolol therapy. Two days post-procedure, the patient reported new-onset, colicky abdominal pain, rated 6/10 VAS in severity, primarily affecting the lower quadrants and radiated to the lower extremities. Associated symptoms included hyporexia, abdominal bloating, and nausea. Physical examination revealed a soft, non-distended abdomen with tenderness only on deep palpation. The pain remitted after a single 1-gram IV dose of acetaminophen. After a 24-h stay, the patient was discharged home. However, four days later, he returned with recurrent abdominal and back pain. CT angiography demonstrated a patent stent in the descending aorta with no evidence of stent fracture, stenosis, aneurysm formation, or vascular dissection.

One month after the aortic repair, follow-up angiography showed continued stent patency with no evidence of leaks or re-stenosis. At the last follow-up, three months post-procedure, the patient remained asymptomatic, and atenolol therapy was discontinued.

## 3 Literature review

**Figure 1:**
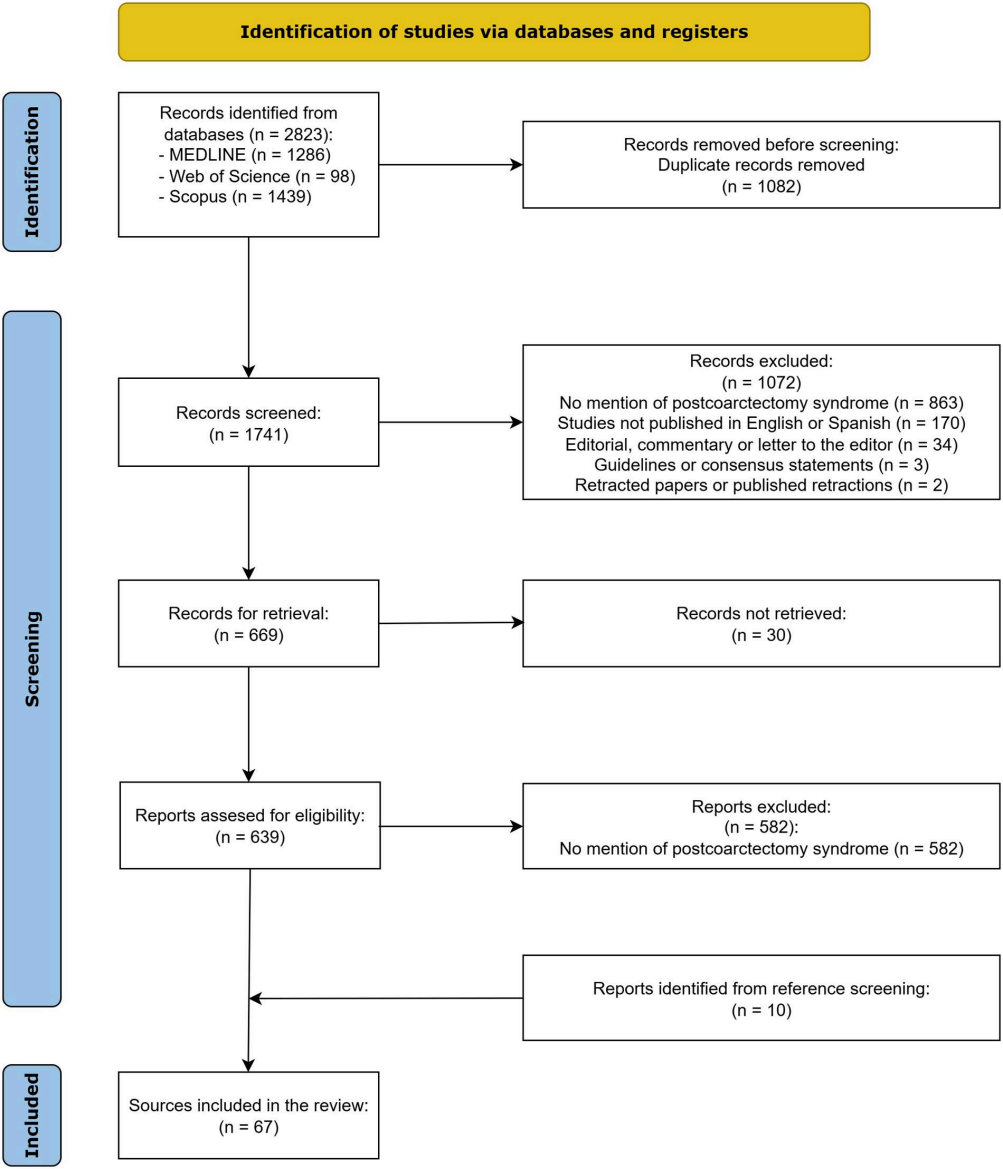
PRISMA flow diagram of study identification and selection.

We conducted a systematic literature review following PRISMA guidelines (9). We performed a comprehensive literature search across PubMed, Web of Science, and Scopus databases for articles published from database inception through July 1st, 2025. Detailed electronic search strategies for each database are provided in Supplementary Table SI. Two reviewers independently screened all identified titles and abstracts for relevance, followed by full-text assessment of potentially eligible studies against predetermined inclusion criteria. Studies were included if they reported on the incidence, pathophysiology, prevention, or treatment of PCS. We excluded editorials, commentaries, studies not specifically addressing PCS, and publications not available in English or Spanish. Any discrepancies between reviewers were resolved through discussion. Reference lists of included studies were screened to identify additional relevant publications. The flow of identified potential sources through the selection process is depicted in Figure 1. Two reviewers independently performed quality appraisal using the JBI Critical Appraisal Tools for clinical studies and the SANRA scale for narrative reviews, depending on each study's design (10–12). Quality assessment results are reported in Supplementary Tables SII-SVI.

## 4 Epidemiology

The reported incidence of PCS varies considerably across studies. The last compilation of incidence data was published in 1965 (3). Given this significant time gap, we sought to provide an updated compilation of reported PCS incidence rates. We identified all available studies reporting either PCS incidence or sufficient data to calculate incidence rates. To avoid case duplication, we cross-referenced authors and institutional affiliations across studies. Our analysis expanded the evidence base from 6 studies in the 1965 review to 37 studies, including two focused on catheter-based interventions, a treatment modality absent from previous reviews (13–42). We also compiled comprehensive symptom and mortality data, which were not detailed in the last compilation. Table 1 summarizes the reported incidence of PCS from these studies. Mortality rate, which ranges from 0% to 3.1% in the largest studies, is detailed in Supplementary Table SVII along with information on reported symptom frequency.

**Table 1:** Reported incidence of postcoarctectomy syndrome.

Report	Publication year	CoA cases operated	Patients surviving	Patients with pcs	Percentage with pcs	Sample demographics
Brom^a^	1965	548	520	16	3.1%	Age: 0–1y (3.2%), 1–10y (19.7%), 10–20y (35.7%), 20–30 (20.9%), 30–60 (20.2%).
Koller et al.^a^	1987	362	362	0	0%	Age: 0–2y (20.4%), 2–19y (39.8%), ≥20y (39.8%).
Ibarra-Perez & Lillehei^a^	1969	Not reported	331	34	10.3%	219 males (66.1%) and 112 females (33.9%).
Lerberg et al.^a^	1982	334	329	32	9.7%	66.6% males, 28% were <1y, and 2.3% were African-american.
Toro-Salazar et al.^a^	2002	274	254	28	11%	Mean age at time of operation was 10.3 ± 9.5 years (median 7.3, range 0 to 41.5). Forty-seven patients were 1 year old at the initial operation, and 31 patients were 20 years old.
Pennington et al.^a^	1979	164	164	4	2.4%	Age: < 1y (9; 5.4%), 1–5y (17; 10.3%), 6–10y (20; 12.1%), 11–18y (42; 25.6%), 19–29y (35; 21.3%), 30–40y (14; 8.5%), > 40y (9; 5.4%).
Clarkson et al.^a^	1983	160	160	9	5.6%	67% males, Age: range 1–54y; 1–9y (40, 25%), 10–19y (62, 39%), 20–29y (26, 16%), 30–39y (16, 10%), 40–19y (11, 7%), ≥ 50 (5, 3%).
Chang & Burrington^a^	1972	194	159	13	8.2%	57 infants, 137 children and adults.
Braimbridge & Yen^a^	1965	119	112	4	3.5%	Mean age 22 years (range 3–56 years).
Palatianos et al.^a^	1985	107	107	0	0%	Mean age 6.4 years (range 4 days – 27 years), 65 males, 42 females.
Stansel et al.^a^	1977	100	99	3	3.0%	Age: 6–12 months (5, 5%), 1–5y (9, 9%), 5–10y (50, 50%), 10–20y (28, 28%), ≥ 20y (8, 8%).
Tawes et al.^a^	1970	103	98	8	8.2%	No details provided
Glancy et al.^a^	1983	70	67	4	5.9%	Mean age 17 years (range 1–49 years), 62 males, 22 females.
Cheatham et al.^a^	1979	80	77	2	2.6%	Mean age 4.5 years (range 1 week to 19 years), 51 males, 29 females. Forty percent were less than 2 years of age, whereas 60% were 2 years of age and older.
Sealy et al.^a^,^b^	1990	71	71	10	14.1%	No details provided
Patel et al.^a^	1977	65	65	5	7.7%	Age range 1–14 years, 30 males, 35 females.
Ring & Lewis	1956	73	64	18	28.1%	Mean age 11 years
Perianayagam et al.^a^	1980	51	46	0	0%	Age range 1 month to 48 years, 35 males and 16 females.
Wittig & Mulder^a^	1980	55	46	1	2.2%	Median age 3.6 months (range 1 day to 12 months), 32 males, 23 females.
Cleland et al.	1956	40	37	5	13.5%	Age range 1–55 years, 27 males, 13 females.
Trummer & Mannix	1963	33	33	8	24.2%	Mean age 11.9 years (range 4–33 years), 23 males, 10 females.
Mays & Sergeant	1965	35	33	4	12.1%	Mean age 10.8 years (range 10 days to 18 years), 22 males and 13 females.
Molaei et al.^c^,^a^	2011	26	26	2	7.7%	Age range 4–19 years.
Fox et al.	1980	25	25	0	0.0%	Age range 1 month to 30 years.
Lindensmith et al.^a^	1971	43	25	3	12%	Age range 2 weeks to 12 months.
Verska et al.^a^	1969	22	22	6	27.3%	Mean age 17.9 years (range 6–46 years), 17 males, 5 females.
Acevedo-Bañuelos et al.^a^	2013	20	20	0	0.0%	Mean age 85 days (range 1–180 days), 13 males, 7 females.
Bergdahl et al.^a^	1980	20	20	0	0%	There were 12 males with a mean age of 43 years (range 35–62 years) and 8 females with a mean age of 44 years (range 37–52 years).
Bergdahl et al.^a^	1980	19	19	1	5.2%	There were 14 boys with a mean age of 10 years (range 5–15 years) and 5 girls also with a mean age of 10 years (range 6–15 years).
Hurt & Hanbury	1957	17	17	2	11.8%	No details provided.
Moreno et al.^a^	1980	17	17	0	0%	Mean age 8.7 ± 5.44 years.
Srouji & Trusler^a^	1965	16	16	6	37.5%	Average age 7.3 years (range 4–15 years).
Reid & Dallachy	1958	15	15	1	6.7%	No details provided.
Parsons & Astley^a^	1966	13	10	2	20%	Age range 6 weeks to 13 years, 8 males, 2 females.
Rocchini et al.^a^	1976	7	7	5	71.4%	Mean age 9.2 years (range 7–14 years), 6 males, 1 female.
Kan et al.	1983	7	7	0	0%	Age range 10 months to 17 years, 6 males, 1 female.
Tefera et al.^c^,^a^	2016	3	3	1	33.3%	Mean age 10 years, 2 males, 1 female.

a Newly added studies since the last incidence compilation reported.

b Updated report comprising the patients of the studies published in 1967 by the same single author.

c Report with patients exclusively treated with catheter-based intervention.

## 5 Risk factors

PCS shows strong male predominance, with males comprising 63–92.1% of cases (3, 36, 43). Large series have reported that, among patients who develop PCS, paradoxical hypertension precedes the syndrome in 25%–100% of cases (3, 14, 16, 26, 36, 40, 41, 44). Age at intervention may be a significant risk factor, with teenagers and adults with facing higher risk than infants (4, 13, 45). This discrepancy may be attributed to age-related factors, including abnormal aortic compliance, altered vasoreactivity, dysfunctional baroreceptors, and up-regulation of the renin-angiotensin system (RAS) (46). Regarding treatment approach, limited data exist comparing PCS incidence between endovascular and surgical repair.

## 6 Pathophysiology

PCS is believed to be the clinical manifestation of necrotizing arteritis that specifically affects vessels located distal to the previously coarcted aortic segment (3, 47–50). However, the exact mechanisms that trigger the development of these vascular lesions remain poorly understood, and multiple competing theories continue to be debated in the literature.

### 6.1 The link with paradoxical hypertension

A central paradigm suggests that PCS shares a common underlying cause with PH, which almost invariably precedes the syndrome (3, 6, 47). PH is a life-threatening, unexpected hemodynamic response that occurs despite successful surgical relief of CoA (39). PH follows a characteristic biphasic pattern. The first phase occurs within 24 h after aortic repair and presents as a brief spike in BP. The second phase develops 48–72 h after coarctectomy and is characterized by severe diastolic hypertension that typically coincides with the onset of the characteristic abdominal pain observed in PCS (22, 39, 51).

### 6.2 Sympathetic nervous system alterations

The elevation of catecholamines following coarctectomy suggests that sympathetic nervous system activation plays a central role in the first phase of PH (42, 52, 53). The mechanism underlying this catecholamine surge appears to be directly linked to altered baroreceptor function. The primary arterial baroreceptors, located in the carotid sinus and aortic arch, are positioned proximal to the coarctation site. Consequently, both structures experience chronic exposure to long-lasting supranormal arterial pressures and gradually reset their regulatory setpoints to accommodate these abnormal hemodynamic conditions (54–56). Following successful coarctectomy, arterial pressure in the proximal aortic segment decreases abruptly. The chronically reset baroreceptors interpret this normal pressure reduction as pathologically low BP. In response, they decrease their inhibitory signals to the bulbar vasomotor centers, which then dramatically increase sympathetic output to compensate for what the altered baroreceptors perceive as hypotension. This compensatory response triggers excessive adrenaline and noradrenaline release (57–59). The elevation of catecholamines after surgery is not unique to coarctectomy, but the magnitude and duration of the noradrenaline surge are considerably greater compared to normotensive or hypertensive patients undergoing other surgical procedures (52, 57).

### 6.3 The role of the renin-angiotensin system

Another possible contributing mechanism involves the postsurgical increase in plasma renin activity and angiotensin II (AT-II). During the second phase of PH, there is a marked increase in both substances. This notable change in hormone concentrations is mediated by increased sympathetic nervous system activity following surgical aortic repair (60). The magnitude and duration of this hormonal response are uniquely characteristic of the postoperative period following coarctation repair (42). The severe abdominal pain and ileus characteristic of PCS may result from excessive mesenteric vasoconstriction induced by these substances, and the ensuing ischemia of the small bowel (3, 5, 38, 49). Given this pathophysiological understanding, RAS antagonists have been proposed as a promising therapeutic approach for controlling postoperative hypertension in this group of patients (61).

### 6.4 The mechanical hypothesis

An additional mechanism contributing to PCS involves the direct mechanical impact of surgical intervention and altered hemodynamics on downstream vasculature. Following aortic repair, the restoration of normal blood flow exposes mesenteric blood vessels to significantly increased pulsatile pressures, potentially causing substantial arterial distension (57). Under these new hemodynamic conditions, the sudden exposure to higher pressures may result in excessive stimulation of viscerosensory fibers within vessel walls and subsequent arterial necrosis. This process culminates in the abdominal pain, distension, and ileus observed in PCS (4, 41, 47). Building on this mechanical theory, it has been suggested that the sudden overdistension of small arteries and arterioles, previously adapted to lower-pressure blood flow, may trigger a protective vasospastic response. While this vasospasm could theoretically protect smaller vessels from further pressure-related damage, it may simultaneously induce bowel ischemia, ultimately causing the gastrointestinal manifestations characteristic of PCS (62, 63).

### 6.5 Experimental evidence for the mechanical hypothesis

The theory that relief of CoA exposes downstream vasculature to increased intra-arterial tension, thereby causing vascular damage, has substantial experimental support. This mechanical explanation for vascular lesions is derived from well-established animal models originally developed to study experimental and malignant hypertension (64–66). Among these experimental studies, Mays & Sergeant (3) highlight the model developed by Byrom & Dodson (66). In this landmark experiment, researchers forcibly injected Ringer's solution into rat carotid arteries, mechanically simulating the sudden increase in intra-arterial tension that occurs following relief of aortic obstruction (66). The resulting arterial lesions were remarkably similar to those described in numerous PCS case reports (8, 38, 55, 56). The experimental results support the concept that intravascular tension approaching the tolerance limits of exposed vasculature can cause overstretching of vessel walls and subsequent necrosis of the muscular layer. These weakened areas provide entry points for blood infiltration, which is then converted to fibrin through thromboplastin released from necrotic muscle cells. This initial damage triggers inflammatory cell infiltration of affected vessels as part of the natural repair process to restore vessel wall integrity (3, 66, 67).

### 6.6 Susceptibility of the mesenteric circulation

An intriguing observation is that, although multiple organs and vascular structures experience similar hemodynamic changes before and after coarctation repair, the splanchnic circulation, particularly the mesenteric vascular territories, demonstrates disproportionately severe involvement (3). The increased susceptibility of mesenteric vessels is theorized to result from two key factors. First, there is a relative lack of surrounding supportive tissue compared to other vascular beds. Second, these vessels possess inherent fragility due to chronic exposure to only subnormal pulse pressures prior to aortic repair (3, 6, 48, 68).

## 7 Prevention

Current strategies to prevent PCS include graduated serial stent dilation, early recognition and treatment of PH, and beta-blocker prophylaxis (45, 54, 69). However, emphasis on beta-blocker prophylaxis has decreased considerably, likely due to significant improvements in postoperative antihypertensive therapy in the ICU since the 1970s (70). This progressive improvement was motivated by the need for alternatives to classic medications such as reserpine and trimethaphan, which were used to manage PH. One of the first major advances was the introduction of non-selective beta-blockers, particularly propranolol, for postoperative PH control (59, 71). Positive results with propranolol increased interest in adrenergic blockers for perioperative BP control in this patient population. Subsequently, esmolol, a selective *β*1-blocker, proved safe and effective for controlling postoperative hypertension in postcoarctectomy patients. Esmolol offered advantages over propranolol, including shorter duration of action and selective adrenoreceptor blockade (72, 73). However, beta-blockers carry a potential disadvantage. Although rare, unopposed beta-blockade with elevated catecholamines can stimulate alpha-adrenergic receptors, potentially causing the mesenteric ischemia characteristic of PCS (58). This limitation has led to recent trials exploring alternative approaches. A retrospective study (46) evaluated the effectiveness of intravenous labetalol, a combined selective *α*1- and non-selective *β*-antagonist, compared to no antihypertensive therapy in pediatric postcoarctectomy patients (46). The study demonstrated that intravenous labetalol was generally safe, effective, fast-acting, and easily convertible to oral therapy when continued treatment was necessary. These findings align with results reported in a small cohort study, which achieved adequate BP control using considerably smaller nitroprusside doses than previously required (58). Despite these advances, treatment approaches remain heterogeneous due to absent standardized guidelines and numerous available pharmacotherapy options (74).

## 8 Treatment

PCS typically resolves following a period of bowel rest and administration of antihypertensive therapy (3, 4, 67). Antihypertensive medications should be initiated immediately upon onset of PH and abdominal pain, using adequate dosages to control both conditions effectively (36). These medications serve dual therapeutic purposes: they reduce mechanical stress on mesenteric arterial walls caused by increased pulsatile blood flow and mitigate the vasoconstrictive and blood-shunting effects of the RAS (6). Additionally, some authors have reported positive outcomes using intravenous papaverine, a non-specific muscle relaxant, as an adjunctive treatment for PCS (5, 75). Supplementary Table SVII provides general information on PCS management as described in sources that documented specific treatments and patient outcomes. Among 121 patients for whom treatment details were reported, symptom duration ranged from 3 to 42 days. Thirteen patients required surgical intervention via laparotomy, and 4 deaths were reported (3, 5, 15, 16, 24, 36, 40, 41, 47, 62, 68, 75). Conservative management represented the most frequently employed therapeutic approach, reported in 75% of studies (3, 5, 15, 16, 41, 47, 62, 68, 75). Conservative measures typically included nil per os, intravenous fluid administration, intubation, and gastric suction. Regarding antihypertensive therapy, reserpine was the most commonly used medication, reported in 30% of studies, followed by hydralazine, which was utilized in 15% of studies (3, 36, 39, 40, 68).

## 9 Discussion

Our understanding of PCS pathophysiology has evolved significantly since early hypotheses were proposed, particularly with advancements in splanchnic circulation physiology. The splanchnic vasculature, including its mesenteric circulation component, is regulated by both intrinsic and extrinsic control mechanisms (76–78). The intrinsic system operates through two primary pathways: metabolic and myogenic. The metabolic pathway induces vasodilation in response to tissue hypoxia and accumulation of metabolic byproducts. The myogenic pathway involves the Bayliss effect, a phenomenon of vasoconstriction that occurs when vascular wall myocytes undergo mechanical stretching. Conversely, decreased intravascular pressure triggers vasodilation, thereby maintaining relatively stable blood flow (78). The extrinsic system encompasses neurohumoral control, autonomic autoregulation, and central cardiovascular control. The neurohumoral component represents a balance between vasoconstrictors such as catecholamines and AT-II, and vasodilators including bradykinin, prostaglandins, and histamine (77, 78). An imbalance in these regulatory systems could explain the clinical findings observed in PCS. As previously discussed, altered barorreceptor setpoints, direct aortic arch manipulation, or both factors can considerably increase circulating catecholamines. These hormones activate *β1*-receptors on the juxtaglomerular apparatus and increase renin secretion, resulting in elevated AT-II levels (79). Both noradrenaline and AT-II produce exaggerated vasoconstriction in the mesenteric vasculature, potentially redistributing up to 80% of splanchnic blood flow and contributing substantially to ischemia in this vascular territory (80). Additionally, increased pulsatile pressure in vessels distal to the repaired coarctation could trigger exaggerated myogenic pathway activation, further reducing blood flow and promoting mesenteric ischemia. This abnormal response appears confined to the mesenteric circulation and may result from differences in adrenoreceptor concentration and subtype ratios between organ systems. Specifically, predominant *α*-mediated vasoconstriction in the mesenteric vasculature could redistribute blood flow away from this territory (80).

Regarding identified knowledge gaps, further research into prevention and treatment of PH is warranted. Building on experiments by Price et al. (81) and findings by Siersma et al. (46) and Charlton et al. (58), prospective randomized studies could compare labetalol with different antihypertensive agents. Such studies should employ pre-specified therapy initiation thresholds, consider higher doses than previously reported, and include metrics such as adverse effect rates, PCS incidence, ICU length of stay, and treatment costs to refine the preventive and therapeutic roles of these medications (82). Similarly, using RAS-antagonists as alternative or adjunct pharmacological management was proposed decades ago, but limited research has explored their therapeutic potential (83).

Notably, patients treated with endovascular techniques appear to have a less prominent sympathetic response, potentially decreasing PH risk (60). This observation warrants further investigation through prospective trials with larger sample sizes and inclusion of PCS incidence as an outcome measure. Additionally, several studies (68)have reported that patients may experience complications even months after coarctectomy (3, 68). Therefore, extended and close follow-up is warranted in future studies to promptly diagnose and address these delayed complications.

The considerable variability in PCS incidence rates across studies may result from differences in patient age, CoA treatment modalities, BP control protocols, and other risk factors that vary significantly between study populations. Future research should investigate how these factors influence PCS incidence rates.

The presented case underscores the need for continued clinical vigilance regarding PCS development. Even with optimal blood pressure management using *β*-blockers and endovascular techniques, this life-threatening complication can emerge days to weeks after CoA repair. Successful management requires rapid recognition and immediate attention to blood pressure control, pain management, and early assessment for potential surgical intervention. Long-term follow-up remains crucial, as patients may develop significant complications following PCS.

## 10 Conclusion

PCS remains an incompletely understood phenomenon with significant clinical implications. This article provides a comprehensive description of current pathophysiological understanding and an updated compilation of incidence data and treatment strategies reported in the literature. Clinical trials investigating novel and complementary pharmacological approaches could substantially improve our understanding of the underlying mechanisms driving PCS and provide a stronger evidence-based foundation for treatment decisions. Given that this complication can be fatal and carries considerable morbidity, healthcare providers treating patients with CoA must remain vigilant throughout the perioperative and extended follow-up periods. Active prevention strategies, prompt recognition and treatment of symptoms, and appropriate long-term follow-up are essential components of care for patients at risk of experiencing PCS. Early intervention and sustained clinical awareness can significantly impact patient outcomes and reduce the morbidity and mortality associated with this serious complication.
