# Supplementary material for: Postcoarctectomy syndrome: a contemporary systematic review
Source: Front Surg. 2025 Nov 17;12:1518720. doi: 10.3389/fsurg.2025.1518720 (PMC12665704; doi:10.3389/fsurg.2025.1518720)
Supplement: Supplementary file 1 [file Table1.docx]

***Supplementary Table I. Electronic search strategies.***

***
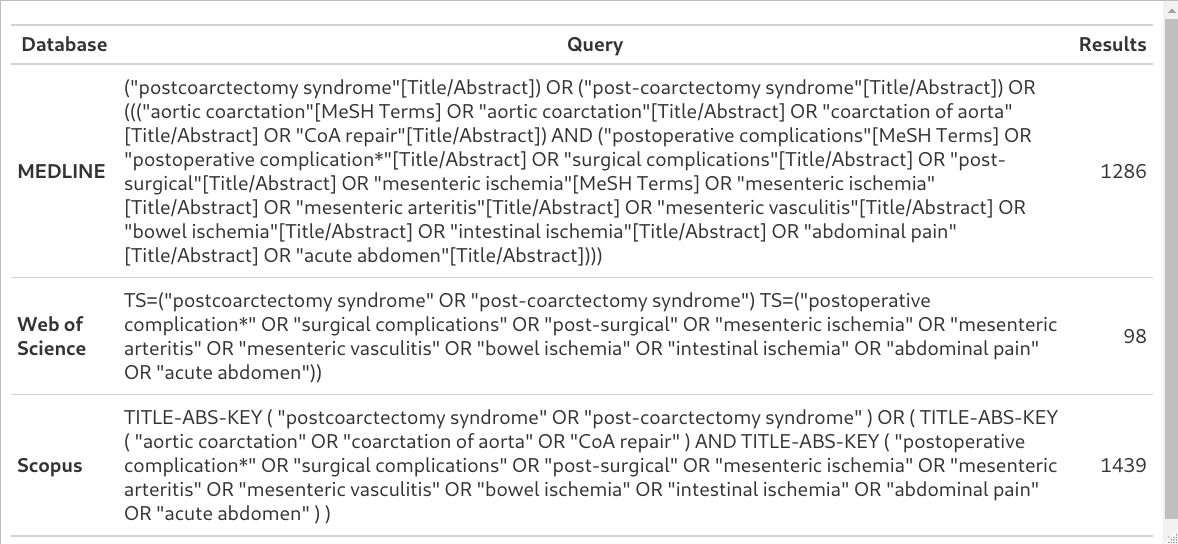
***

***Supplementary Table II. Quality appraisal for included case reports.***


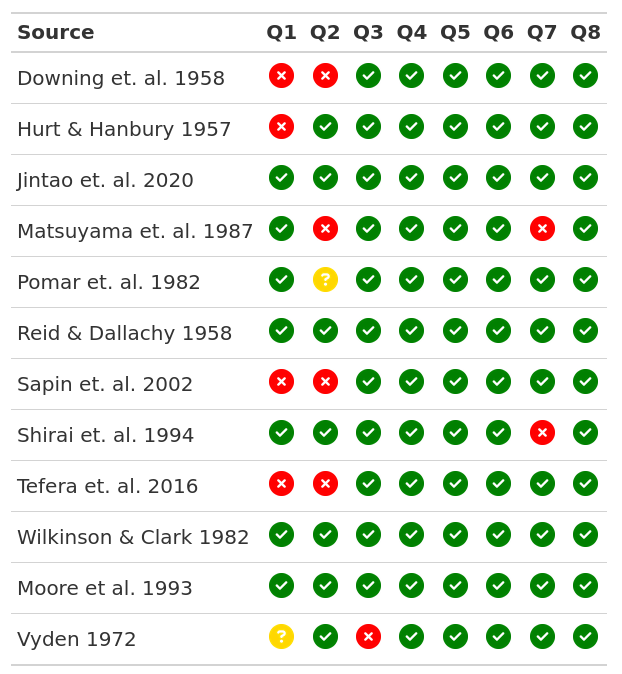


Yes:
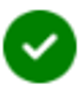
 No:
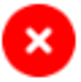
 Unclear:
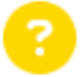


***Supplementary Table III. Quality appraisal for the included case-control study.***


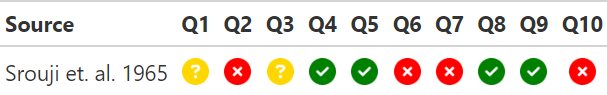


Yes:
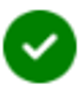
 No:
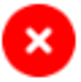
 Unclear:
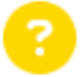


***Supplementary Table IV. Quality appraisal for included cohort studies.***


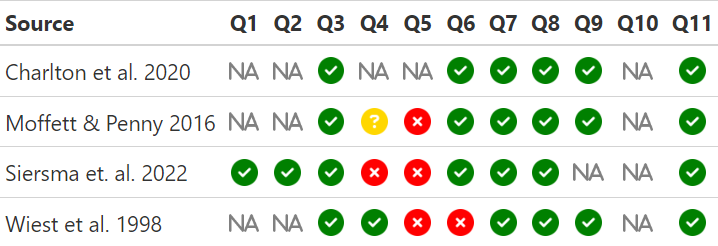


Yes:
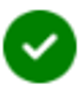
 No:
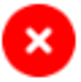
 Unclear:
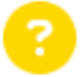
 Not applicable:
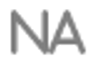


***Supplementary Table V. Quality appraisal for included case series.***


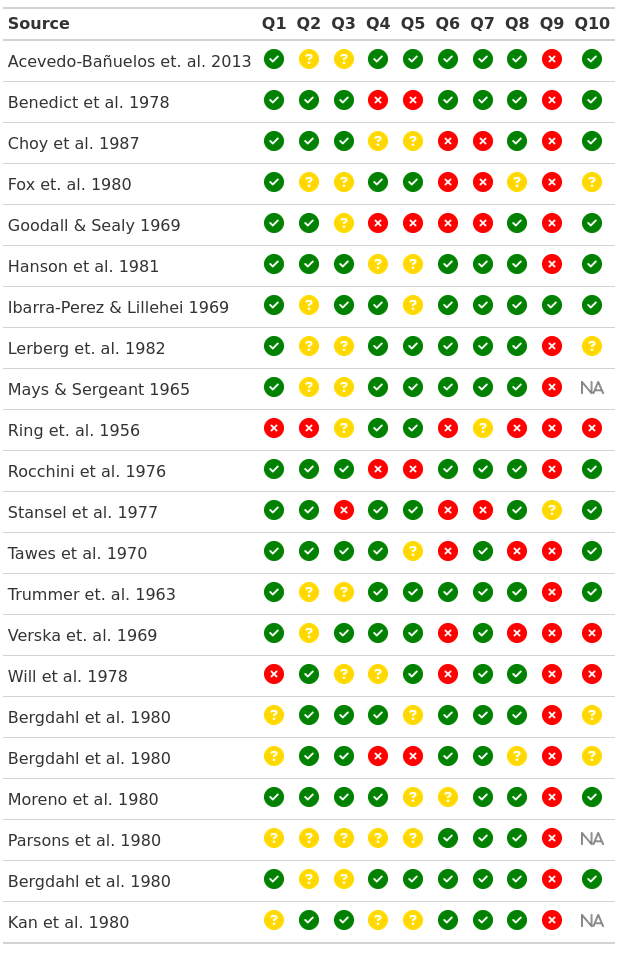
Yes:
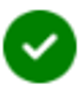
 No:
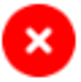
 Unclear:
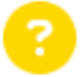
 Not applicable:
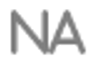


***Supplementary Table VI. Quality appraisal for included narrative reviews.***

***
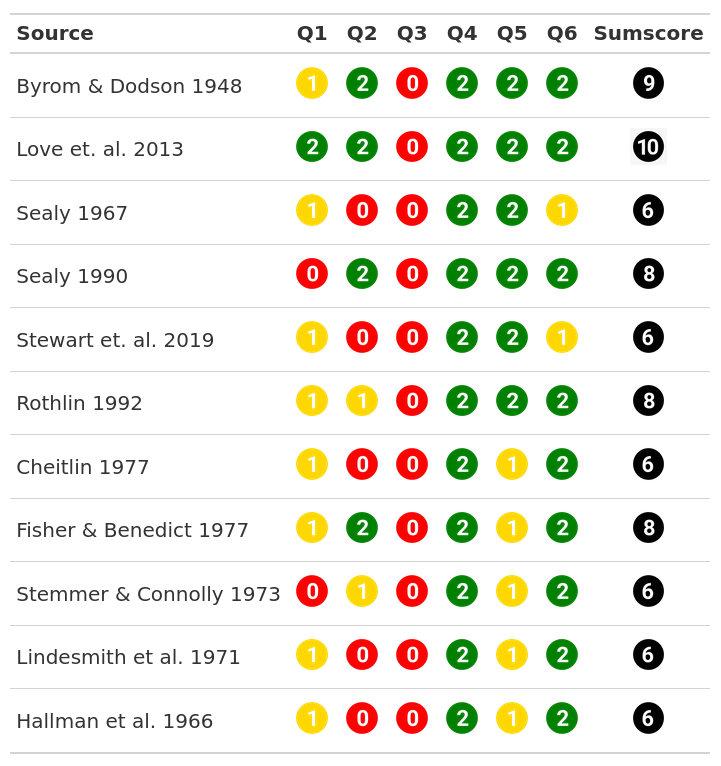
***

***Supplementary Table VI. Reported Treatments for PCS***

| **Source** | **Patients with PCS** | **Presentation** | **Symptom onset** | **Symptom duration** | **Rate of laparotomies** | **Treatment** | **Outcome** |
| --- | --- | --- | --- | --- | --- | --- | --- |
| Ibarra-Perez & Lillehei 1969 | 34 | Abdominal pain, tenderness, rebound, GI bleeding, abdnormal bowel sounds, fever, leukocytosis, ileus on X-ray | Postoperative days 1-11 | Mean of 4.4 days with treatment vs 7.8 without treatment | 2/34 | Hydralazine, reserpine, phenoxybenzamine, trimethaphan camphorsulfonate | 2 deaths |
| Ring et. al. 1956 | 18 | Abdominal pain, abdominal distention, gastric dilatation, abdominal tenderness, ileus, or vomiting, leukocytosis, fever | Postoperative days 1-10 | 7-10 days | 4/18 | Nil per os, gastric suction, IV fluids, meperidine, methadon | Response to conservative therapy |
| Trummer et. al. 1963 | 8 | Abdominal pain, distention, leukocytosis, left-upper quadrant periumbilical tenderness, rigidity, rebound tenderness, hypoactive or absent bowel sounds, and evidence of free intraperitoneal fluid | Postoperative days 1-6 | 5-17 days | 0/8 | IV or oral reserpine, intestinal intubation and suction, intravenous fluid and electrolyte replacement, and systemic antibiotics. | Abdominal pain disappeared and appetite returned |
| Sealy 1990 | 6 | Abdominal pain, leukocytosis | Postoperative days 3-4 | Mean of 10 days in laparotomy patients | 2/6 | IV hydralazine or reserpine | 2 patients with laparotomy died. The 4 patients with antihypertensives had a prompt symptom resolution |
| Verska et. al. 1969 | 6 | Abdominal pain, distention, fever, and leukocytosis | After 48 hours postintervention | Up to 7 days | 0/6 | Reserpine and Hydralazine | Relief of symptoms |

***Supplementary Table VI. Reported Treatments for PCS (Continued)***

| **Source** | **Patients with PCS** | **Presentation** | **Symptom onset** | **Symptom duration** | **Rate of laparotomies** | **Treatment** | **Outcome** |
| --- | --- | --- | --- | --- | --- | --- | --- |
| Srouji et. al. 1965 | 6 | ''Moderate and severe clinical features of the syndrome.'' No details provided | After 2nd postoperative day | No details provided | 0/6 | Conservative treatment | All responded well |
| Mays & Sergeant 1965 | 4 | Abdominal pain, distension, rigidity, melena, fever, vomiting | Postoperative day 2-4 | 8 to 42 days | 1/4 | Reserpine, nasogastric intubation, parenteral alimentation | 1 death |
| Stansel et al. 1977 | 3 | Abdominal pain, fever, leukocytosis, melena | No details provided | No details provided | 0/3 | Conservative treatment | All responded quickly and none experiencied any sequelae |
| Jintao et. al. 2020 | 1 | Diffuse abdominal distention and hypoactive bowel sounds | 12 hours after stent placement | 6 days | 0/1 | Nil per os, IV urapidil and papaverine | Abdominal pain disappeared on the 6th day |
| Reid & Dallachy 1958 | 1 | Colicky abdominal pain, vomit, tenderness, muscle guarding, leukocytosis | 11th Postoperative day | 5 days | 1/1 | Nil per os, IV fluids | Negative clinical examination, no delay nor obstruction of small bowel, episodes of severe abdominal pain since discharge |
| Tefera et. al. 2016 | 1 | Severe abdominal pain, vomiting | 12 hours after procedure | 36 hours | 0/1 | Potent analgesics, prolongation of oral feeding restriction, and antihypertensive management intensification | The patient was stabilized within 48 hours following the procedure |

***Supplementary Table VI. Reported Treatments for PCS (Continued)***

| **Source** | **Patients with PCS** | **Presentation** | **Symptom onset** | **Symptom duration** | **Rate of laparotomies** | **Treatment** | **Outcome** |
| --- | --- | --- | --- | --- | --- | --- | --- |
| Pomar et. al. 1982 | 1 | Severe abdominal pain, abscence of peristaltic sounds | Postoperative days 6 | 18 hours | 0/1 | Gastric drainage, intraarterial catheterization of the superior mesenteric artery with infusion of papaverine, IV nitroprusside and metyldopa. | Resolution of abdominal symptoms within 18 hours of treatment initiation |
| Moore et al. 1993 | 1 | Melena, distended abdomen with decreased bowel sounds, tachycardia and hypotension. | Postoperative day 2 | 4 days | 0/1 | Over the next 4 days he was treated with intravenous fluids and antibiotics, and he was given nothing by mouth. | On day 6 after the procedure, feedings were started and were well tolerated. After two additional uneventful days the patient was discharged to home. |
| Clarkson et al. 1983 | 9 | No details provided. | No details provided. | No details provided. | 2/9 | Two patients underwent laparotomy but neither required bowel resection. | Both patients that underwent laparotomy recovered. |
| Bergdahl et al. 1980 | 1 | Abdominal pain, meteorism and vomiting. | Postoperative day 7 | No details provided | 1/1 | Gastrostomy and feeding jejunostomy. | The patient died 1 month later, during an abdominal surgery, |

***Supplementary Table VI. Reported Treatments for PCS (Continued)***

| **Source** | **Patients with PCS** | **Presentation** | **Symptom onset** | **Symptom duration** | **Rate of laparotomies** | **Treatment** | **Outcome** |
| --- | --- | --- | --- | --- | --- | --- | --- |
| Wittig & Moulder 1980 | 1 | No details provided | No details provided | No details provided | 0/1 | Reserpine | The patient responded well to reserpine treatment. |
| Cheatham et al. 1979 | 2 | Ileus and abdominal pain | No details provided | No details provided | 0/2 | No details provided | No details provided |
| Pennington et al. 1979 | 4 | Abdominal pain, tenderness and distention, hematuria, and prolonged ileus | No details provided | 3 to 10 days | 0/4 | IV chlorpromazine + trimethaphan and methyldopa + propranolol. | None of these four patients had residual bowel symptoms at the time of hospital discharge. |
| Chang & Burrington 1972 | 13 | Abdominal pain, fever, leucocytosis, and melena | No details provided | No details provided | 0/13 | No details provided | No details provided |
| Vyden 1972 | 1 | Severe abdominal pain of a colicky nature, fever, leukocytosis, and diffuse abdominal tenderness with increasing abdominal distention. Serial abdominal radiographs showed cecal dilatation and increasing multiple, small, fluid levels in the small intestine. | Postoperative day 6 | 3 days | 0/1 | Methicillin, 1 Gm. four times per day IM, and streptomycin, 1 Gm. twice a day IM. | On the ninth hospital day, the patient's pain, abdominal swelling, and hypertension subsided. |

***Supplementary Table VII. Reported symtom frequency and mortality for PCS patients.***

| **Report** | **Symptom frequency** | **PCS-related mortality** |
| --- | --- | --- |
| Brom, 1965 | Not reported. | No deaths associated to PCS. |
| Koller et al., 1987 | No patients developed PCS. | No patients developed PCS. |
| Ibarra-Perez & Lillehei, 1969 | Abdominal pain (100%), tenderness (82.3%), rebound (38.2%), GI bleeding (14.7%), abnormal bowel sounds (75.4%). | 1/34 PCS patients died (2.94%). |
| Lerberg et al., 1982 | Not reported. | 1/32 PCS patients died (3.12%). |
| Toro-Salazar et al., 2002 | Not reported. | No deaths associated to PCS. |
| Pennington et al., 1979 | One patient had only mild abdominal cramps and distention lasting 72 hours. Two severe cases were characterized by abdominal pain, tenderness and distention, hematuria, and prolonged ileus lasting at least 10 days after operation. | No deaths associated to PCS. |
| Clarkson et al., 1983 | Not reported. | No deaths associated to PCS. |
| Chang & Burrington, 1972 | Abdominal pain (100%), fever (23%), leukocytosis (23%), melena (23%). | No deaths associated to PCS. |
| Braimbridge & Yen, 1965 | Not reported. | No deaths associated to PCS. |
| Palatianos et al., 1985 | No patients developed PCS. | No patients developed PCS. |
| Stansel et al., 1977 | Abdominal pain (100%), fever (100%), mild leukocytosis (100%), melena (33%). | No deaths associated to PCS. |
| Tawes et al., 1970 | Not reported. | No deaths associated to PCS. |
| Glancy et al., 1983 | Abdominal pain (100%). | No deaths associated to PCS. |
| Cheatham et al., 1979 | Ileus and abdominal pain (100%). | No deaths associated to PCS. |
| Sealy et al., 1990 | Insufficient information to calculate frequency of symptoms. | No deaths associated to PCS. |
| Patel et al., 1977 | Abdominal pain (100%) and melena (40%). | No deaths associated to PCS. |
| Ring & Lewis, 1956 | Insufficient information to calculate frequency of symptoms. | No deaths associated to PCS. |

| **Report** | **Symptom frequency** | **PCS-related mortality** |
| --- | --- | --- |
| Perianayagam et al., 1980 | No patients developed PCS. | No patients developed PCS. |
| Wittig & Mulder, 1980 | Abdominal pain (100%) and ileus (100%). | No deaths associated to PCS. |
| Cleland et al., 1956 | Abdominal pain (100%), massive ascitic effusion (20%). | No deaths associated to PCS. |
| Trummer & Mannix, 1963 | Abdominal pain (100%), vomiting (100%). | No deaths associated to PCS. |
| Mays & Sergeant, 1965 | Abdominal pain (100%), distention (100%), nausea (100%), and vomiting (100%), melena (50%), leukocytosis (50%). | 1/4 PCS patients died (25%). |
| Molaei et al., 2011 | Transient abdominal pain (100%). | No deaths associated to PCS. |
| Fox et al., 1980 | No patients developed PCS. | No patients developed PCS. |
| Lindensmith et al., 1971 | Severe abdominal pain (100%) and ileus (100%). | No deaths associated to PCS. |
| Verska et al., 1969 | Insufficient information to calculate frequency of symptoms. | No deaths associated to PCS. |
| Acevedo-Bañuelos et al., 2013 | No patients developed PCS. | No patients developed PCS. |
| Bergdahl et al., 1980 | No patients developed PCS. | No patients developed PCS. |
| Bergdahl et al., 1980 | Abdominal pain (100%), meteorism (100%), and vomiting (100%). | 1/1 PCS patients died (100%). |
| Hurt & Hanbury, 1957 | Insufficient information to calculate frequency of symptoms. | No deaths associated to PCS. |
| Moreno et al., 1980 | No patients developed PCS. | No patients developed PCS. |
| Srouji & Trusler, 1965 | Four children (66%) developed moderate and two children (33%) developed severe clinical features of PCS. | No deaths associated to PCS. |
| Reid & Dallachy, 1958 | Abdominal pain (100%), vomiting (100%). | No deaths associated to PCS. |
| Parsons & Astley, 1966 | Abdominal pain (100%), melena (50%), dyspnea (50%), paresthesia (50%). | No deaths associated to PCS. |
| Rocchini et al., 1976 | Insufficient information to calculate frequency of symptoms. | No deaths associated to PCS. |
| Kan et al., 1983 | No patients developed PCS. | No patients developed PCS. |
| Tefera et al., 2016 | Severe abdominal pain (100%), vomiting (100%). | No deaths associated to PCS. |
